# Targeting HDACs for diffuse large B-cell lymphoma therapy

**DOI:** 10.1038/s41598-023-50956-x

**Published:** 2024-01-02

**Authors:** Chunyan Wu, Qiao Song, Sophie Gao, Shaoling Wu

**Affiliations:** 1 Department of Hematology, The Affiliated Hospital of Qingdao University, No.16 Jiangsu Road, Qingdao, 266003 Shandong China; 2 Department of Hematology, People’s Hospital of Jiyang District, Jiyang, 251400 Shandong China; 3 Hunter College High School, New York, USA

**Keywords:** Lymphoma, Cell death, Cell growth

## Abstract

Histone deacetylases (HDACs) are involved in tumorigenesis and progression, however, their role in diffuse large B-cell lymphoma (DLBCL) is not well understood. In this study, we examined the expression levels, mutations, and clinical significance of HDACs in DLBCL. Additionally, we investigated the therapeutic potential of Chidamide, a novel HDAC inhibitor, to provide scientific evidence for targeting HDACs in DLBCL patients. We extracted transcriptome data of DLBCLs––including 47 lymph node samples and 337 whole-blood-cell controls––from The Cancer Genome Atlas. Bioinformatic analyses of HDAC expression, mutation, and correlation with the clinical significance of DLBCL patients were performed with the Gene Expression Profiling Interactive Analysis, GENEMANIA, and web-based software including cBioPortal and WebGestalt. To examine the therapeutic effect of Chidamide, DLBCL cell lines (WSU-DLCL-2 and DB cells) were employed. Cell proliferation and apoptosis were analyzed with Cell Counting Kit-8 and flow cytometry assays. The impact of Chidamide treatment was also analyzed by RNA sequencing of treated DB cells. Western blot was used to explore the molecular mechanism of the cytotoxicity of Chidamide on DLBCL cell lines. The expression of some HDACs (HDAC1, 2, 3, 4, 6, 7, 8, and 9) were significantly higher in the lymph node samples of DLBCL than that in whole-blood-cell controls. Moreover, we found that the mutation rate of HDACs was also higher in DLBCL tissues, although the overall survival of DLBCL patients was not associated with HDAC expression. Chidamide was found to have a cytotoxic effect on DLBCL cells in a dose-dependent manner, while transcriptome analysis and western blot revealed that using it for treatment impacted several biological processes, including PI3K/AKT signaling, mTOR signaling, the cell cycle, and apoptosis pathways. Alterations of HDAC genes, including enhanced expression and mutations, are positively related to DLBCL. Targeting HDACs with specific inhibitors such as Chidamide may represent a potential therapeutic approach for DLBCL patients.

## Introduction

Diffuse large B-cell lymphoma (DLBCL) is the most common lymphoma, accounting for 23–25% of non-Hodgkin's lymphoma. Although R-CHOP immunochemotherapy, a combination therapy using rituximab, cyclophosphamide, doxorubicin, vincristine, and prednisone, can cure approximately 60–65% of DLBCL patients, the remaining 35–40% often have poor outcomes, including relapse or refractory disease^1,2^. There is, then, an urgent need to better understand the molecular mechanisms and identify accurate prognostic biomarkers in DLBCL to improve its management and treatment.

Histone deacetylases (HDACs) are enzymes that regulate gene expression by removing acetyl groups from histones and other proteins^3^. They play a critical role in processes like development, cellular differentiation, and cellular homeostasis^4,5^. HDACs are divided into four classes^6^ based on their structure, mechanism of action, and cellular localization: Class I (HDAC1, HDAC2, HDAC3, HDAC8), Class IIa (HDAC4, HDAC5, HDAC7, HDAC9), Class IIb (HDAC6, HDAC10), and Class IV (HDAC11). Dysregulation of HDAC activity has been implicated in various diseases, including neurodegenerative disorders, genetic diseases, and cancer^7–9^. In cancer, abnormal expression of HDACs is associated with poor prognosis. It has been linked to the development and progression of various types of cancer, including hematologic malignancies such as acute lymphoblastic leukemia (ALL), acute myeloid leukemia (AML), and chronic myeloid leukemia (CML)^6^.

Inhibition of HDACs has emerged as a promising therapeutic strategy for the reatment of cancer and other diseases thanks to their role in inducing changes in gene expression and cellular behavior. These changes may ultimately lead to growth inhibition, apoptosis, and differentiation of cancer cells. As a result, HDAC inhibitors are being evaluated in various clinical trials for their efficacy in the treatment of cancer.

Chidamide is a selective inhibitor of Class I and II HDACs^10–13^, including HDAC1, HDAC2, HDAC3, and HDAC10, and has been approved for the treatment of peripheral T-cell lymphoma (PTCL) in China since 2015^14^. Chidamide was found to be an effective treatment for relapsed or refractory DLBCL with an acceptable safety profile^15^. The study also showed that Chidamide was effective as a monotherapy for relapsed or refractory DLBCL, with an overall response rate (ORR) of 25% and a complete remission rate (CR) of 15%. Chidamide could treat DLBCL by causing apoptosis in DLBCL cells by inhibiting the HDACs/STAT3/Bcl2pathway^16^ or inhibiting cells with mutant TP53^10^.

This study aims to investigate the expression, mutations, and clinical significance of HDACs in DLBCL, as well as the potential therapeutic benefits of targeting HDACs with Chidamide. Our aim is to provide scientific evidence to support clinical decision-making and risk management in DLBCL patients.

## Materials and methods

### Reagents

Chidamide was kindly provided by CHIPSCREEN BIOSCIENCES (Shenzhen, China). The agent was dissolved in dimethyl sulfoxide (DMSO, Sigma, MO, USA) to obtain a stock solution of 50 mM, and stored at − 80 °C.

### Cell lines and culture

Human B-lymphoma cell line DB was obtained from Procell Life (Wuhan, China). WSU-DLCL-2 was obtained from Byeotime (Chengdu, China). DB cells and WSU-DLCL-2 cells were maintained in Roswell Park Memorial Institute-1640 (RPMI-1640; Procell, Wuhan, China) supplemented with 10% fetal bovine serum (FBS; Procell, Wuhan, China), 100 U/ml penicillin, and 100 mg/ml streptomycin. Cells were cultured in a humidified incubator with 5% CO_2_ at 37 °C.

### Cell viability assay

Cells (2 × 10^4^ cells/ well) were seeded into 96-well plates and treated with Chidamide with a series of concentrations as described for 24 or 48 h. Cell viability was analyzed by using the Cell Counting Kit-8 (CCK‑8, Yeasen, Shanghai, China ) with absorbance at 450 nm measured by a microplate reader (Tecan Group Ltd., Switzerland).

### Cell apoptosis assay

Cells (1.5 × 10^5^ cells/well) were seeded into 6-well plates and treated with Chidamide as described. They were collected and stained by Annexin V and propidium iodide (PI) (Yeasen, Shanghai, China) following a standardized protocol. Cell apoptosis was analyzed by flow cytometry (FACSCalibur; BD Biosciences, USA).

### Gene expression profiling interactive analysis (GEPIA)

GEPIA2 (http://gepia2.cancer-pku.cn/) is an online database that integrates large amounts of data from The Cancer Genome Atlas (TCGA) and the Genotype-Tissue Expression project (GTEx) to analyze multidimensional cancer genomics^17^. It was used to compare gene expression levels between DLBCL and normal tissue using ANOVA and produce scatter diagrams and box plots. Patient survival analysis was conducted using Kaplan–Meier curves for further verification. It was used to compare gene expression levels between DLBCL and normal tissue using ANOVA and produce scatter diagrams and box plots. Additionally, patient survival analysis was conducted using Kaplan–Meier curves for further verification.

### cBioPortal

cBioPortal (http://www.cbioportal.org/) is a user-friendly web interface used to analyze gene variations in DLBCL^18^, including amplifications, mutations, and copy number variations. It also provides an overview of the genetic alterations of each HDAC family member and detailed information on each type of mutation in individual samples.

### Correlation analyses

Correlation between every two HDACs was assessed using a Pearson’s correlation coefficient. Statistical analysis and the graph were finished with R (v3.6.3). *P* < 0.05 was considered significant.

### GENEMANIA and WebGestalt

To investigate potential interactions among the genes, we used the GENEMANIA website (http://genemania.org/) to perform a gene network analysis^19^. Gene Ontology (GO) and Kyoto Encyclopedia of Genes and Genomes (KEGG) enrichment analysis was conducted using the WEB-based Gene SeT Analysis Toolkit (WebGestalt, http://www.webgestalt.org/)^20^. The GO terms were divided into three categories: biological processes (BP), cellular component (CC), and molecular function (MF). The results of the GO, KEGG, and Reactome analysis were plotted using the ggplot2 package in R (v3.6.3).

### LinkedOmics database

We used the online LinkedOmics database (http://www.linkedomics.org/) to identify the most relevant genes of each HDAC family member^21^. The top 50 genes that were significantly associated with HDACs were displayed in a heatmap and a volcano plot.

### RNA sequencing analysis

The DB cells were cultured with or without Chidamide (5 µM) for 48 h. Total RNA was extracted, and a sequencing library was constructed and sequenced using the Illumina platform. RNA-seq data was analyzed by BMKCloud(www.biocloud.net). Differential gene expression analysis was conducted using DESeq 2. Genes that had a Fold Changed ≥ 1.5 and q-value < 0.05 were considered as significant the differentially expressed genes(DEGs). Pearson's correlation coefficient determined the correlation between samples. Gene Ontology (GO) enrichment analysis of the DEGs was performed using the cluster Profiler package based on Wallenius non-central hyper-geometric distribution, which accounts for gene length bias in DEGs. KEGG pathway enrichment of the DEGs was assessed using the KOBAS database and cluster Profiler software.

### Western blot analysis

The cells were inoculated into a culture flask and treated with Chidamide for 48 h. To extract the total proteins, cell lysates (Elabscience, Wuhan, China) were used, and the samples were quantified using a bicinchoninic acid (BCA) kit (Elabscience, Wuhan, China). Equal amounts of protein were separated by sodium dodecyl sulfate–polyacrylamide gel electrophoresis (SDS-PAGE) and then transferred to a polyvinylidene difluoride (PVDF) membrane (Millipore, Billerica, MA, USA). A primary antibody was incubated with the PVDF membrane overnight at 4 °C after being sealed with 5% skim milk. After washing steps, the PVDF membrane was incubated with a secondary antibody for 70 min and finally detected with a chemiluminescence substrate. An endogenous internal control for protein loading was the relative expression of GAPDH.

### Statistical analysis

All experiments were repeated at least three times. Data were analyzed using GraphPad Prism 9.3.1 (GraphPad Software, USA) and are presented as the mean ± standard deviation of at least three separate experiments. Correlation between HDACs was determined using Pearson's correlation coefficient. Statistical analysis and graphs were created using R (v3.6.3). *P*-value < 0.05 was considered as significant correlation. One-way analysis of variance (ANOVA) was used to compare cell viability, cell proliferation, and different time points and concentrations in DB and WSU-DLCL-2 cells. *P*-value < 0.05 was considered statistically significant.

## Results

### Histone deacetylase (HDAC) genes in diffuse large B-cell lymphoma (DLBCL)

**Figure 1:**
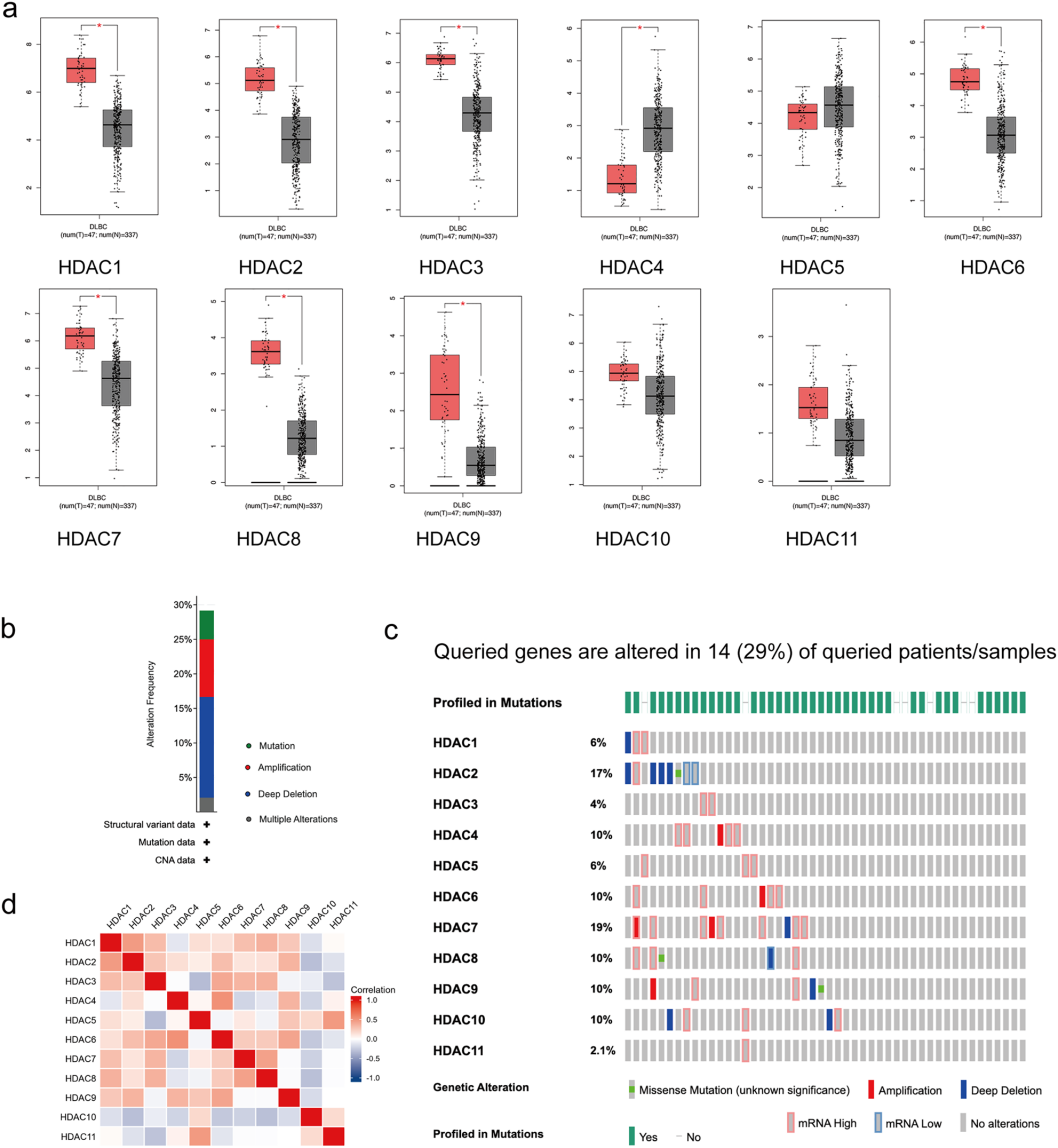
HDAC genes alterations in DLBCL. (**a**) Box plots show the differential expression of HDACs in DLBCL. (**b**) The Mutation frequency of HDACs in DLBCL. Percentages of mutation, amplification, deep deletion or multiple alterations are shown in different colors. (**c**) The mutation rates of each HDAC family member highlighting the significant amplifications and/or mRNA high of HDACs in DLBCL. (**d**) The correlation of gene alterations between two HDACs.

To investigate the changes in gene expression and mutations of HDACs in relation to DLBCL, we used multiple bioinformatic tools, including GEPIA and cBioPortal.We analyzed 47 lymph node samples from DLBCL patients and 337 whole-blood-cell samples from controls, obtained from the TCGA database. The results revealed that the expression of HDACs, including HDAC1, HDAC2, HDAC3, HDAC4, HDAC6, HDAC7, HDAC8, and HDAC9, were significantly elevated compared to normal tissue controls (Fig. 1a), while the other HDACs (HDAC5, HDAC10, and HDAC11) showed no significant difference. Using cBioPortal tools, we discovered a high rate of alteration (29%) of HDACs in DLBCL samples (Fig. 1b,C). Notably, the most frequent alterations include amplification and/or high mRNA in HDAC3, HDAC4, HDAC5, HDAC6, and HDAC7 (Fig. 1c), which indicated their elevated expression. In addition, the Pearson correlation analysis revealed significant positive correlations between HDAC family members, especially HDAC1, HDAC2, HDAC7, and HDAC8 (Fig. 1d). Then, the limited clinical data available to us did not allow us to establish a significant correlation between HDAC expression levels and overall survival of DLBCL patients (Fig. S1). However, our findings demonstrated that the Kaplan–Meier survival curves for HDAC1 and HDAC8 were clearly separated after around 50 months, respectively. This suggests that HDAC1 and HDAC8 may have 50 months or even longer impacts on the survival of tumor patients.

Together, these bioinformatic analyses suggest an overall elevated expression of HDACs in DLBCL and indicate that explicitly targeting HDAC activities may benefit DLBCL patients.

### Functional enrichment and pathway analysis of HDAC family

**Figure 2:**
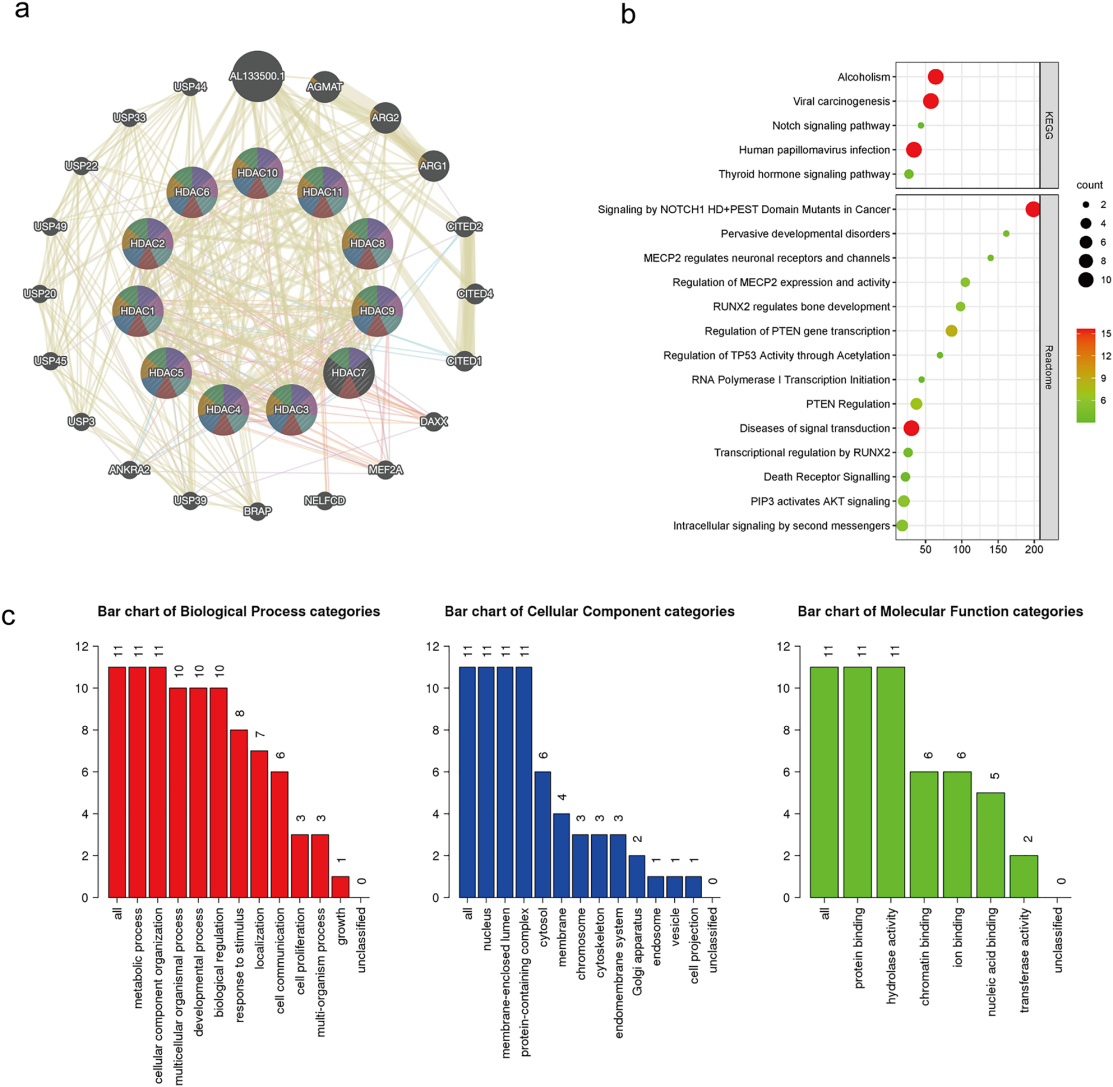
The biological functions of HDAC-interacting proteins. (**a**) Protein–protein interaction (PPI) network of HDACs. (**b**) WebGestalt assay of HDAC-interacting proteins with KEGG pathways. (**c**) Enrichment of biologic pathways of HDAC-interacting proteins. Each biological process, cellular component, and molecular function category is represented by red, blue, and green bars, respectively. The height represents the enriched number of IDs in the pathway.

To understand the biological role of HDACs, we analyzed their protein interactions using GeneMANIA and found the proteins that most interacted with HDACS to include ALT133500.1, AGMAT, ARG2, ARG1, CITED2, CITED4, and CITED1. These proteins are associated with functions such as macromolecule deacetylation, hydrolase activity, protein deacetylation, and deacetylase activity (Fig. 2a). To further explore the downstream functions of the HDAC-interacting proteins, we performed Gene Ontology (GO) and Kyoto Encyclopedia of Genes and Genomes (KEGG) pathway analyses using WebGestalt. Interestingly, in addition to show casing the expected protein binding and chromosome-related pathways, the KEGG pathway enrichment analysis also revealed that important module genes were mainly enriched in the Notch signaling pathway, which is related to malignancy development (Fig. 2b). The GO term analysis showed that the BP categories of important modules mainly included CC organization, metabolic process, and multicellular organismal process; The CC categories included nucleus, membrane-enclosed lumen and protein-containing complex; and the MF categories mainly included protein-binding, ion binding and hydrolase activity (Fig. 2c).

Furthermore, we analyzed genes that are co-expressed with HDACs in DLBCL patients using the LinkedOmics database (Fig. S2).The most correlated genes, positive or negative (Table 1), would indicate the specific biologic functions of HDACs in DLBCL.

**Table 1:** HDACs co-expressed genes in DLBCL.

	Positive related	Negative related
HDAC1	GDA2, KHDRBS1, FAF1	LRRC33, MAP3K5, MLKL
HDAC2	CENPW, HSF2, IFT52	ASPDH, LRRC69, APOL2
HDAC3	SUEDC2, ST20, FPGS	EDEM3, ZNF638, MYST3
HDAC4	AAK1, AGAP1, APOL6	BTF3, MCTS1, C21orf57
HDAC5	INPP5K,TADA3,SLC38A10	WDR43,MRPL19, MPHOSP10
HDAC6	NXT2, 2NF182, GRIPAP1	N6AMT2, MIF, BRF2
HDAC7	TCF3, LASS4, ISHROOM1	SLFN12L, THEMIS, TRAT1
HDAC8	KAP, MED8, RBM10	HPS4, CRAMP1L, ZMYM2
HDAC9	LCO441204,RMND5A,CCDC88C	RABEPK, PTPRW, PPA2
HDAC10	ABCD4, C22orf40, CHKB	RAB23, TNPO1, GAPVD1
HDAC11	SMPD1, TTLL1, BCL7C	DNA2,HAUS6,RAD51AP1

### Cytotoxic effects of Chidamide in DLBCL cells

The correlation of HDAC expression, mutation, and function with DLBCL prompted us to further examine the cytotoxic effect of HDAC inhibitors in DLBCLs. We selected Chidamide, a specific inhibitor of Class I and II HDACs, including HDAC1, HDAC2, HDAC3, and HDAC10, which showed a significant association with DLBCL in our bioinformatic analyses.

**Figure 3:**
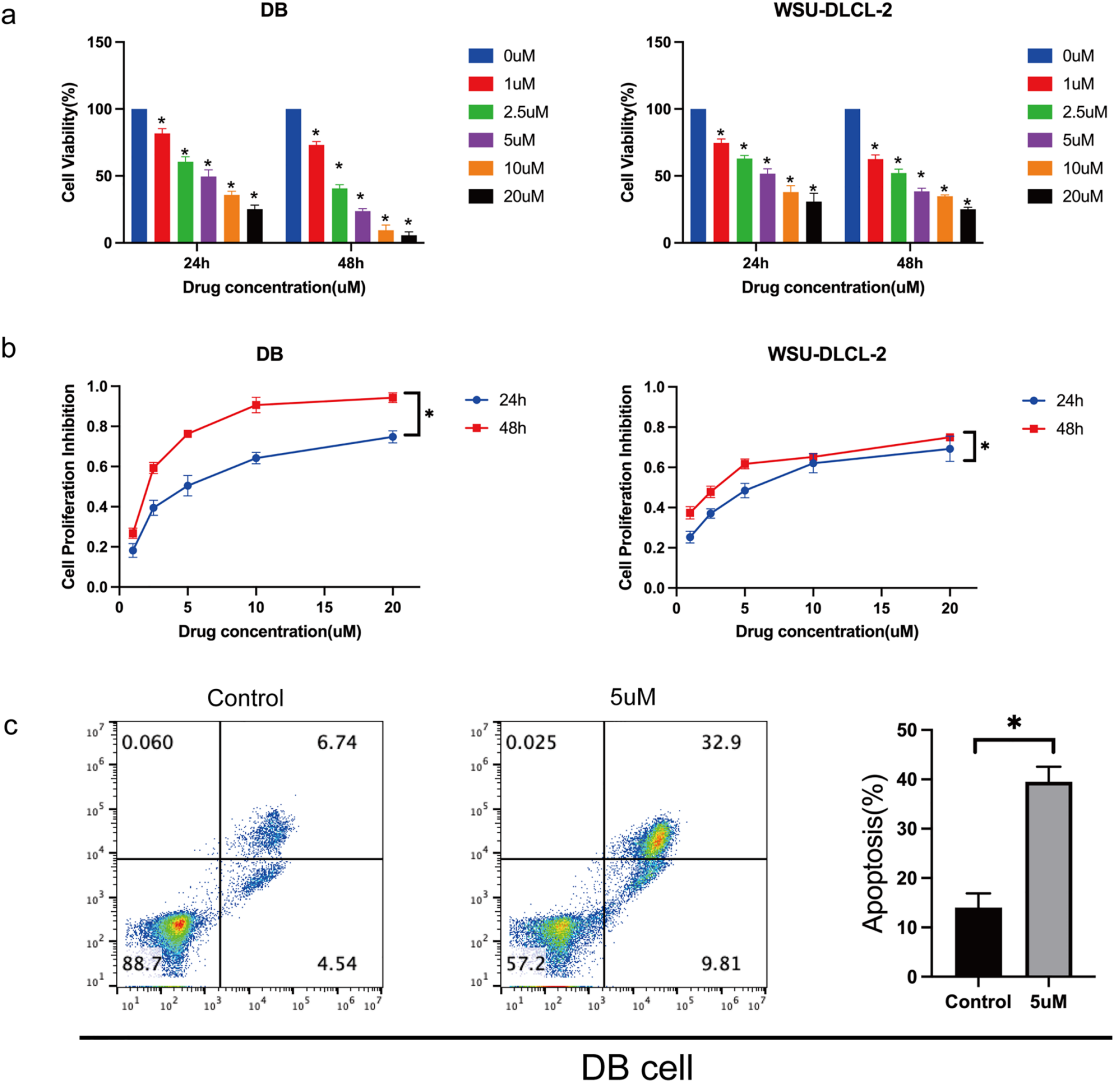
Cytotoxic effects of Chidamide in DLBCL cells. a,b WSU-DLCL-2 and DB cells were treated with an array of concentrations of Chidamide (1, 2.5, 5, 10 or 20 μmol/L) for 24 or 48 h. Cell viability was measured by CCK-8 assay (**a**, **b**) and flow cytometry (**c**). *P < 0.05.

DLBCL cells (DB and WSU-DLCL-2) were seeded and treated with various concentrations of Chidamide (1, 2.5, 5, 10, 20 μM) for 24 and 48 h. The cytotoxic effect was measured using the CCK-8 method. Consistently, Chidamide inhibited proliferation in a dose-dependent manner in both cell lines (Fig. 3a,b). Flow cytometry assays also revealed a significant increase in apoptotic cells in the cells treated with Chidamide compared to controls treated with DMSO (Fig. 3c). These results suggest the therapeutic potential of HDAC inhibitors in DLBCL.

### Effects of HDAC inhibitor Chidamide on transcriptomes of DLBCL cells

**Figure 4:**
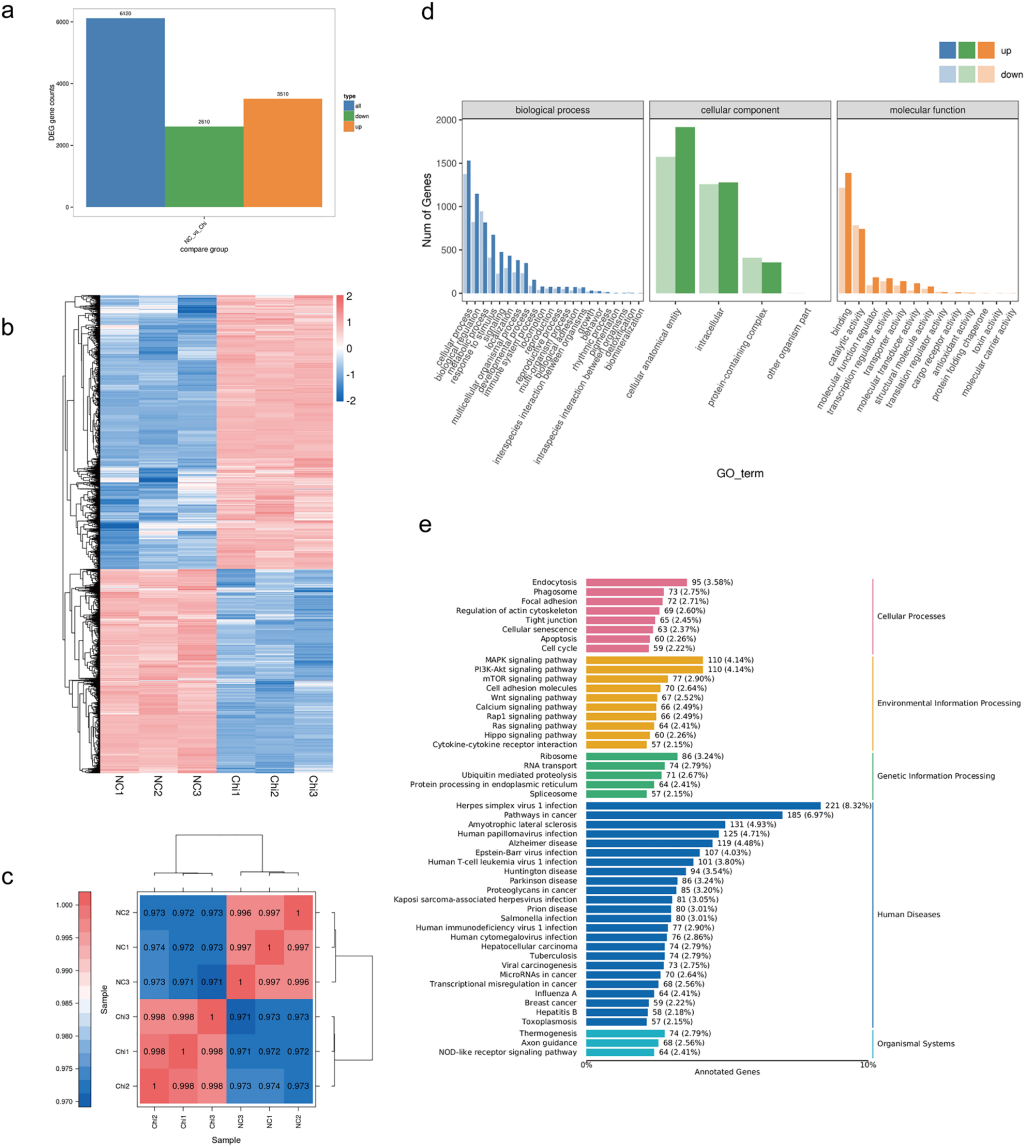
Transcriptional analysis of DB cells treated with Chidamide. (**a**) Bar graph shows the total number of differentially expressed genes (3510 upregulated genes and 2610 downregulated genes) in Chidamide-treated cells compared to DMSO-treated controls. (**b**) Heatmap of differentially expressed genes. (**c**) Correlation between every two samples was assessed using a Pearson’s correlation analysis. (**d**, **e**) Pathway enrichment assay of differentially expressed genes with GO term and KEGG pathways.

Next, we studied the specific impact of HDAC inhibitors on the transcriptome of DLBCL by performing an RNA-sequencing analysis. Compared to the untreated control cells, we found that a total of 6,120 genes were differentially expressed genes in Chidamide-treated DB cells (3510 genes up-regulated and 2610 genes down-regulated, Fig. 4a.b). We used Pearson's Correlation Coefficient (r) as an index for assessing biological replicate correlation, and the closer the calculated r2 was to 1, the stronger the correlation between the two replicate samples (Fig. 4c). Also of note was how Chidamide did not alter the expression of most HDACs, including HDAC1, HDAC2, HDAC3, and HDAC10, suggesting that it works by inhibiting deacetylation functions in HDACs instead of limiting their expression directly.

With the differential expression genes, we further analyzed their related biological functions by a combined GO term and KEGG pathway enrichment assay. We found that Chidamide impacted crucial biological processes such as the cell cycle and apoptosis pathways, which is consistent with our cytotoxic assay (Fig. 4d). Notably, Chidamide treatment also significantly impacted genes related to MAPK signaling, PI3K/AKT signaling, mTOR signaling, and Wnt signaling, suggesting potential mechanisms for DLBCL treatment with Chidamide (Fig. 4e).

### Effects of Chidamide on c-Myc, BCL2, and TP53 proteins in DLBCL

**Figure 5:**
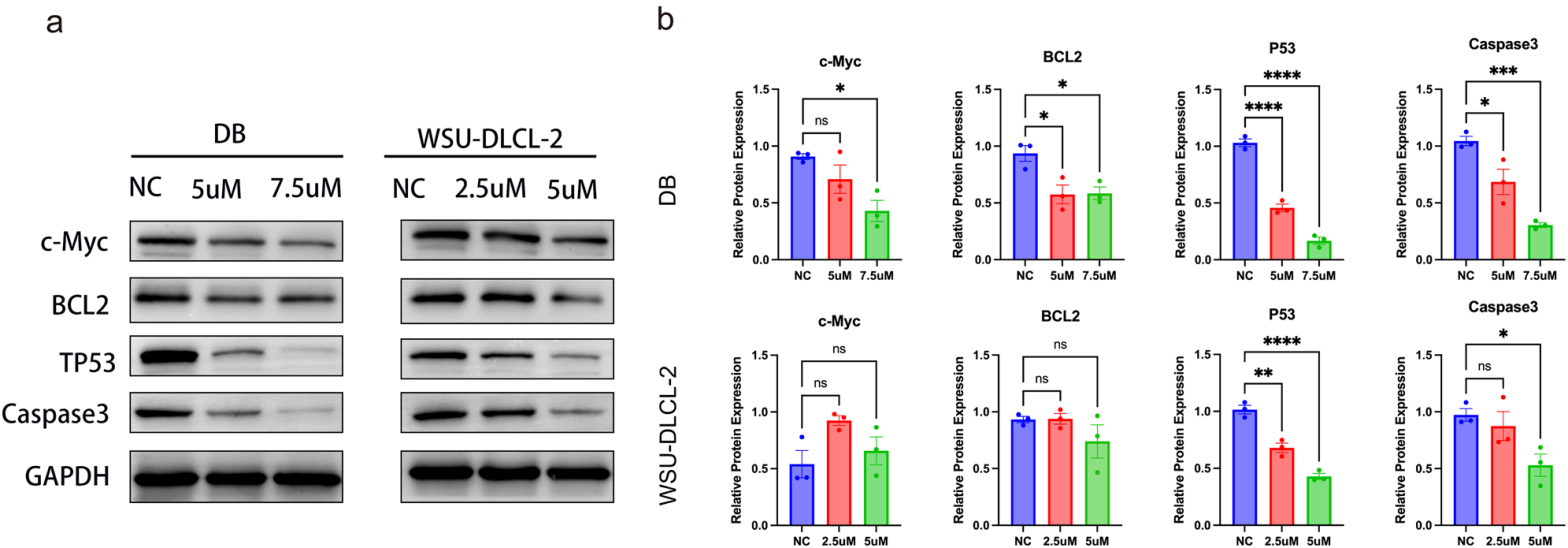
(**a**) Chidamide treatment of DB or WSU-DLCL-2 cells for 48 h. The proteins c-Myc, TP53, BCL2, and Caspase3 were measured by western blot. (**b**) Quantitative analysis of the western blot results in a. Image J was used to analyze the gray value changes in c-Myc, TP53, BCL2, and Caspase3. GAPDH was used as the internal control. Data are presented as the mean ± SEM. Compared with the control group, *P < 0.05, **P < 0.01, ***P < 0.001, ****P < 0.0001.

WSU-DLCL-2 and DB cell lines were treated with Chidamide for 48 h to observe changes in c-Myc, BCL2, and TP53 protein levels in response. The expression of c-Myc, BCL2, and TP53 is associated with the prognosis of DLBCL, and the increase of their expression may indicate a poor prognosis^22–24^. As shown in (Fig. 5a,b), the expression of TP53 and the apoptotic protein Caspase 3 was down-regulated. As the concentration of Chidamide increased, the down-regulation of TP53 and Caspase 3 protein became more pronounced compared with the control group (*P* < 0.05). BCL2 and c-Myc protein levels were dramatically reduced in DB cells, while there was no discernible trend in WSU-DLCL-2. The studies showed that Chidamide could induce apoptosis and further suppress the levels of c-Myc, BCL2, and TP53.

### Chidamide interfering with the PI3K/AKT/mTOR signaling pathways in DLBCL cells

**Figure 6:**
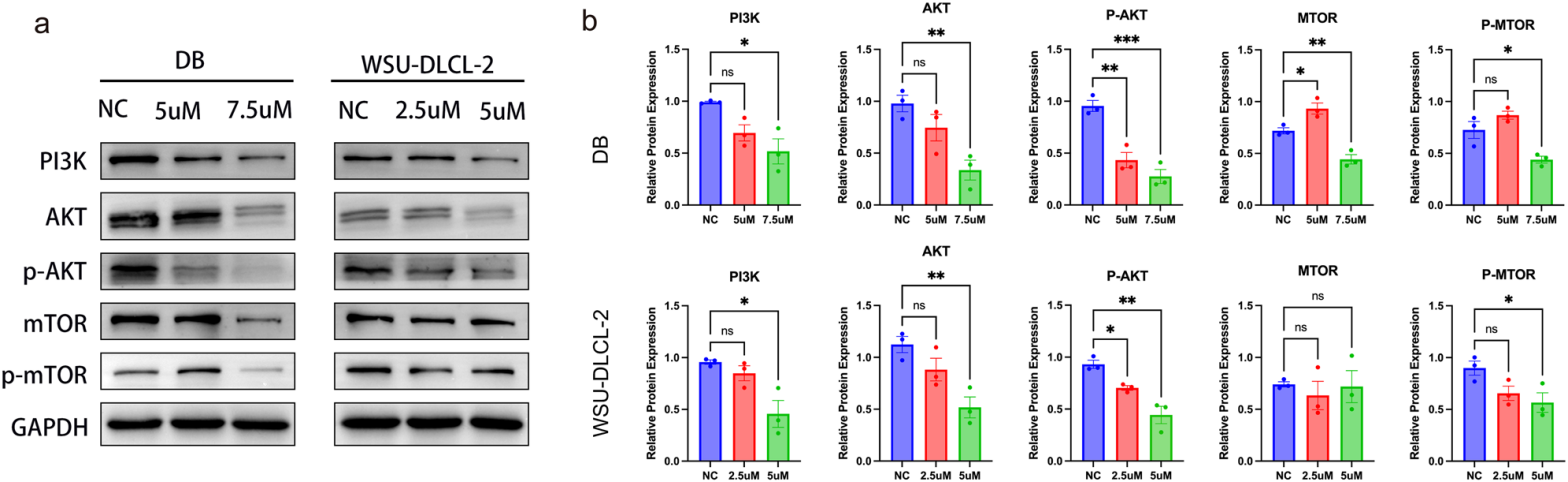
Chidamide promotes apoptosis by altering proteins in the cancer pathway. (**a**) Western blot of PI3K/AKT and mTOR signaling proteins in DB and WSU-DLCL-2 cells. (**b**) Statistical analysis of relative gray values of protein (PI3K (p85), AKT, p-AKT, mTOR, p-mTOR) bands (n = 3). GAPDH was used as the internal control. Data are presented as the mean ± SEM. Compared with the control group, *P < 0.05, **P <  0.01, ***P < 0.001.

To address the molecular mechanism of the effects of Chidamide in DLBCL cells, and we next performed western blot to characterize the protein level changes in different DLBCL cell lines after treatment. As shown in (Fig. 6a,b), we first selected the relatively sensitive cell lines DB and WSU-DLCL-2. The results showed that the protein levels of PI3K(p85), AKT, mTOR, and phosphorylated AKT(Ser473), phosphorylated mTOR(S2448) were significantly decreased. Our sequencing results highlighted the PI3K/AKT and mTOR signaling pathways as potential targets. Similarly, Chidamide downregulated the protein levels of PI3K and p-AKT, thus intensifying the inhibitory effect on the mTOR pathway. This further modulates proteins such as c-Myc, BCL2, TP53, and Caspase3, ultimately promoting apoptosis in DLBCL cells.

## Discussion

In our comprehensive analysis of HDACs in DLBCL patients using public databases, we found that the expression levels of HDAC1, HDAC2, HDAC3, HDAC6, HDAC7, HDAC8, and HDAC9 were higher in tumor tissues compared to normal control for the first time. However, there was no statistical significance of overall survival for DLBCL patients with high HDAC expression compared to those with low expression. A possible explanation of these observations is that HDACs may play a critical role in the tumorigenesis stage of DLBCL, as reported in other studies.

**Figure 7:**
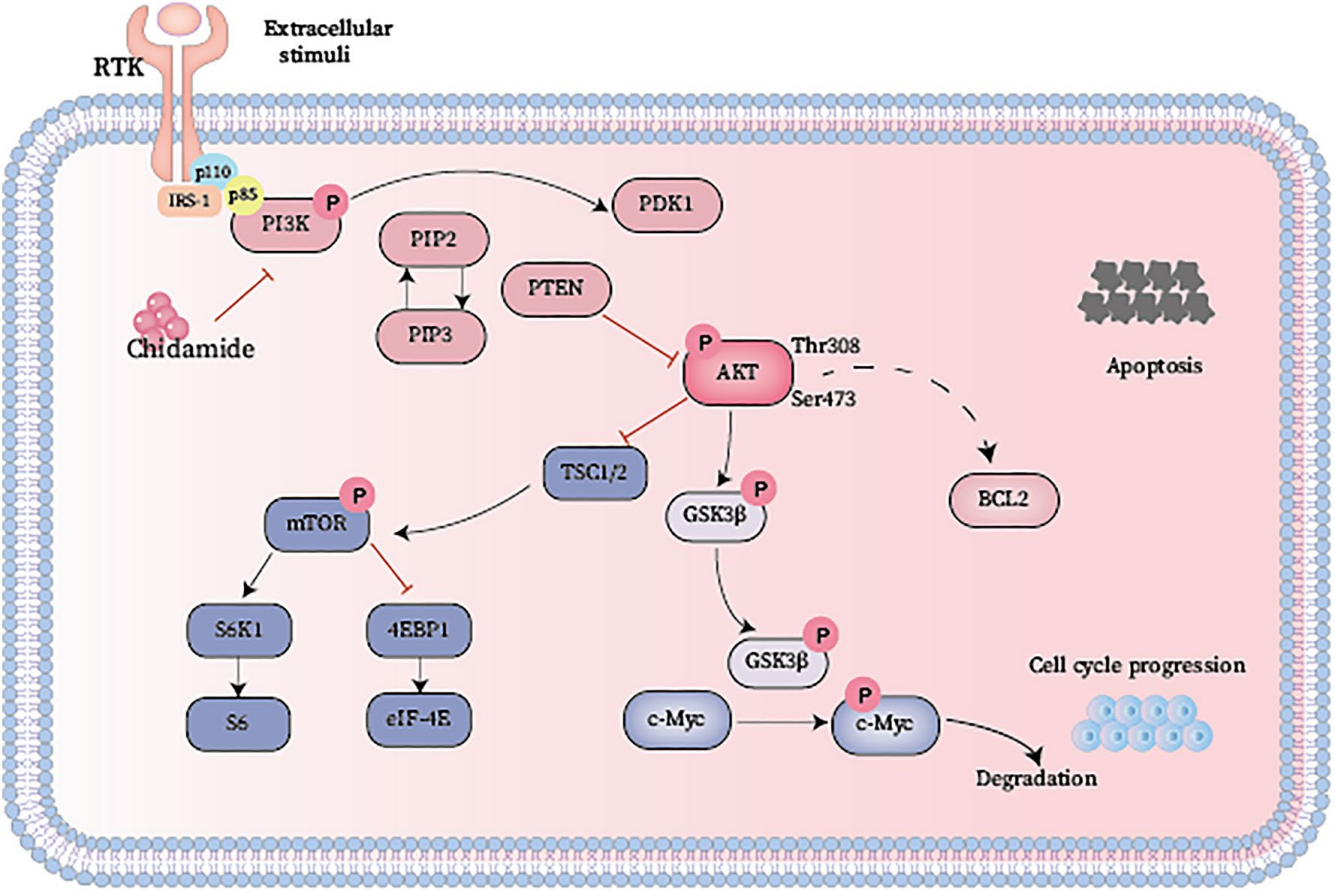
Mechanism of Chidamide treatment. A schematic representation of the PI3K/AKT/mTOR pathway.

Chidamide, a novel inhibitor of histone deacetylases, can suppress the Class 1 HDACs HDAC1, HDAC2, HDAC3, and HDAC8 in DLBCL cell lines^16^. Consistently, our study found that exposure of WSU-DLCL-2 and DB cells to Chidamide for 24–48 h led to dose and time-dependent inhibitions of cell viability, possibly through induction of apoptotic cell death. Transcriptome analysis and western blot demonstrated that Chidamide treatment had an impact on a variety of biological processes, including PI3K/AKT signaling, mTOR signaling, the cell cycle, and apoptosis pathways. The genesis and progression of several illnesses, including cancer, as well as normal physiological functions, are significantly influenced by the PI3K/AKT/mTOR signaling pathway^25^. Simultaneously, it is considered one of the most common pathways for tumor inactivation^26^. Under ordinary physiological conditions, it controls cellular processes, including proliferation, mitosis, and metabolism via its downstream effectors, 4EBP1 (4E binding protein) and S6K1 (S6 kinase beta1)^27^. Aberrant activation of the PI3K/AKT/mTOR pathway in pathological situations causes disruptions to cellular proliferation, which in turn causes angiogenesis to be amplified, drug resistance to rising, and cell growth to accelerate^28^. The PI3K/AKT/mTOR signaling pathway plays a significant role in B-cell growth and development^29^, and in lymphomas, this pathway exhibits heightened activity. This pathway is frequently dysregulated in cancer, with increased PI3K/AKT signaling being linked to various activities, including tumor cell proliferation, apoptosis suppression, and promotion of invasion and metastasis^30^ (Fig. 7).

Among the HDAC family members, HDAC1 has been well characterized and may indicate a poor prognosis in DLBCL cases^31^. Its overexpression has been correlated with poor prognosis in diffuse large B-cell lymphoma^32^. Chidamide, as a selective inhibitor of HDAC1 and HDAC2, may be a treatment option for DLBCL patients with HDAC1 and HDAC2 overexpression.

Moreover, HDAC3 is a crucial regulator of PD-L1 transcription in B-cell lymphomas, and its inhibition increases the response to anti-PD-1 therapy^33^. Targeting HDAC3 may be a promising approach for B-cell lymphoma immunotherapy.

Rituximab is a chimeric monoclonal antibody targeted against the pan-B-cell marker CD20. A subset of patients develop CD20 expression deficiency after Rituximab therapy, which may lead to Rituximab retreatment failure^34^. Insufficient surface CD20 protein affects lipid raft domain organization and downstream signaling, leading to Rituximab resistance^35^. Chidamide significantly increased CD20 surface expression in DLBCL cell lines, which is a promising sensitizer for the retreatment of DLBCL with Rituximab^36^. It is that Histone deacetylation causes gene silencing and inhibits CD20 expression deficiency, is a major obstacle to retreatment of relapsed/refractory DLBCL with rituximab-related regimens, inhibition of HDAC6 activity considerably enhances CD20 levels in established B-cell tumor cell lines and primary malignant cells^37^.

Additionally, specific targeting of other HDACs, including HDAC5 and HDAC8, has been shown to be effective in the treatment of T-cell lymphomas and neuroblastomas^38^. New selective HDAC inhibitors are expected to be available for DLBCL patients in the near future.

In conclusion, our study suggests that HDACs may play a critical role in the diagnosis and treatment of DLBCL and that targeting them with inhibitors such as Chidamide may be a promising therapeutic approach. Further studies are needed to validate the therapeutic potential of HDAC inhibitors in DLBCL patients.

### Supplementary Information

Supplementary Figure 1.

Supplementary Figure 2.

Supplementary Information 3.

Supplementary Legends.

## Data Availability

Some databases that support the findings of this study is openly available and described in “Materials and methods”, including GEPIA2^17^, cBioPortal^18^, GENEMANIA website^19^, and WebGestalt^20^. LinkedOmics database^21^. TCGA (https://portal.gdc.cancer.gov) and GETx datasets (https://www.gtexportal.org/home/datasets). Other data are available from the corresponding author.
