# Supplementary figures and images for: Targeting HDACs for diffuse large B-cell lymphoma therapy
Source: Sci Rep. 2024 Jan 2;14:289. doi: 10.1038/s41598-023-50956-x (PMC10762105; doi:10.1038/s41598-023-50956-x)

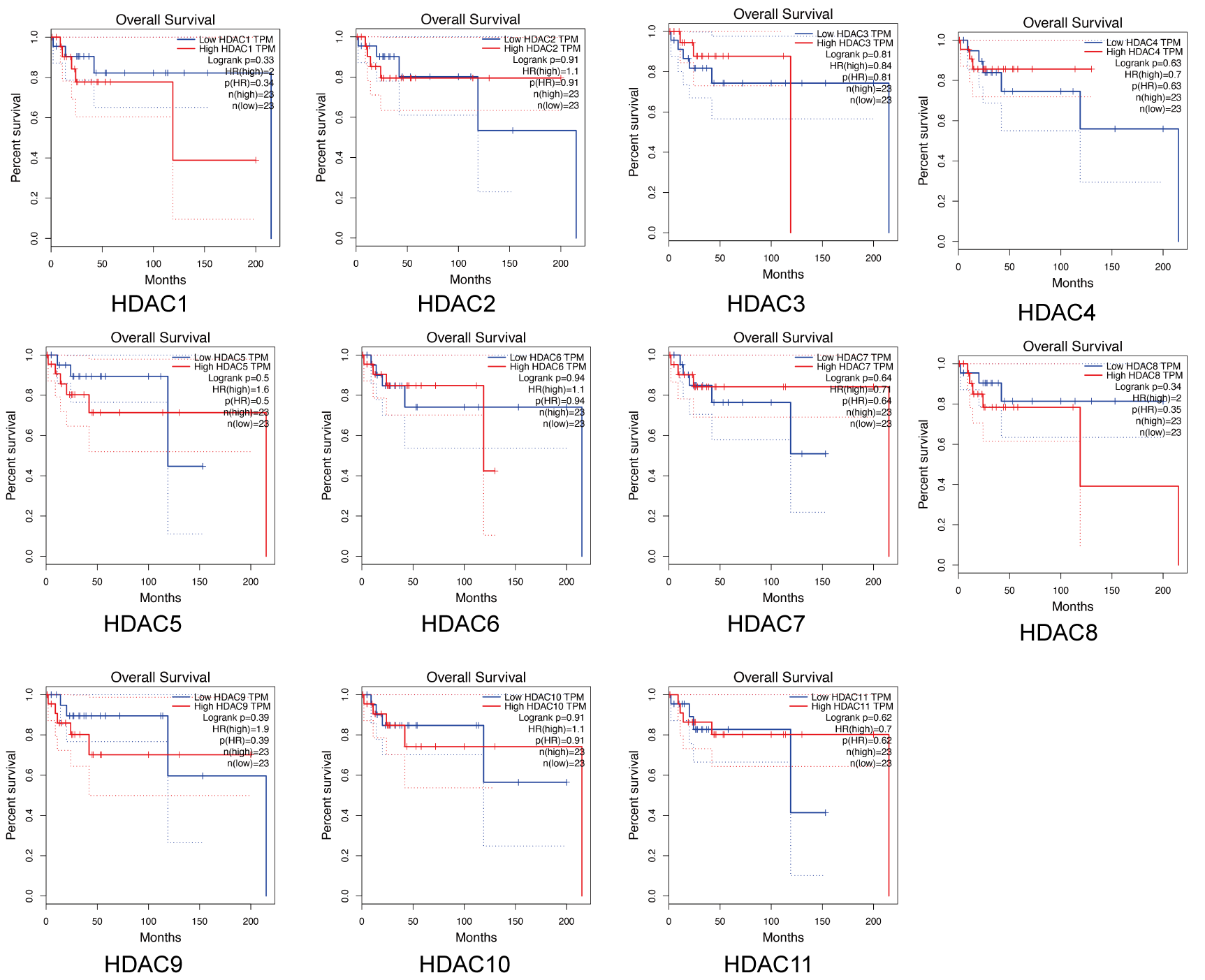

Supplement: Supplementary file 1 — Supplementary Figure 1. [file 41598_2023_50956_MOESM1_ESM.tif]

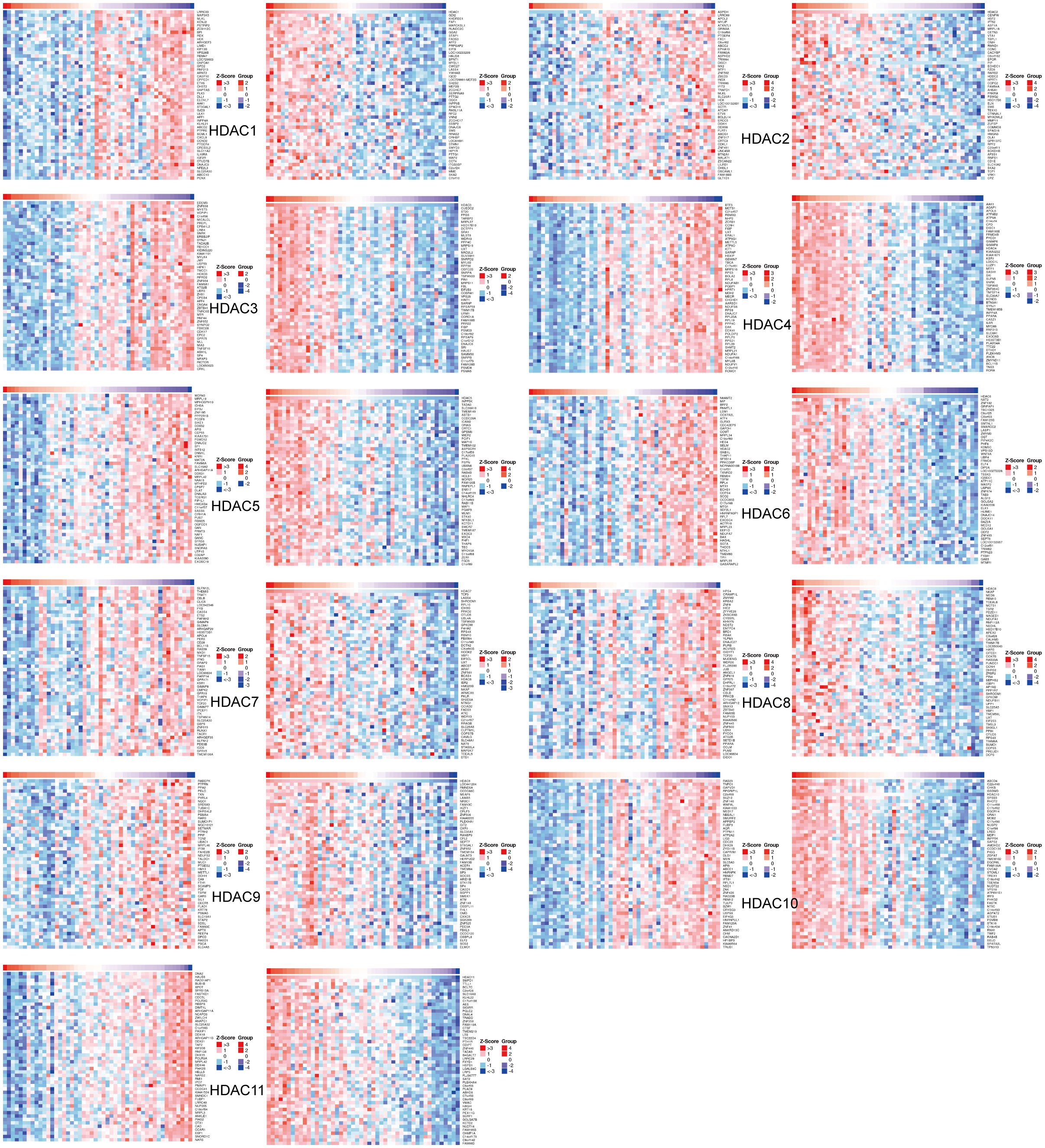

Supplement: Supplementary file 2 — Supplementary Figure 2. [file 41598_2023_50956_MOESM2_ESM.tif]
