# Supplementary material for: Targeting HDACs for diffuse large B-cell lymphoma therapy
Source: Sci Rep. 2024 Jan 2;14:289. doi: 10.1038/s41598-023-50956-x (PMC10762105; doi:10.1038/s41598-023-50956-x)
Supplement: Supplementary file 3 — Supplementary Information 3. [file 41598_2023_50956_MOESM3_ESM.pdf]

Original, complete and unchanged photographs of our western blots

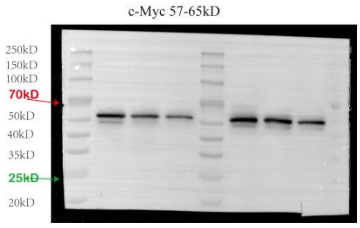

C-MYC

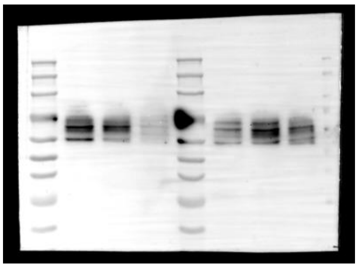

C-MYC

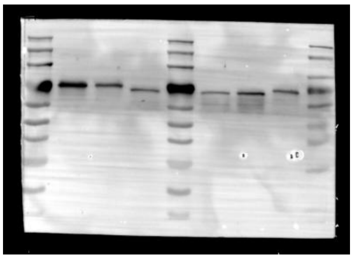

C-MYC

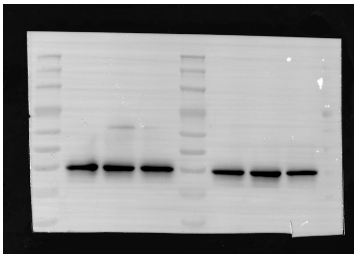

GAPDH

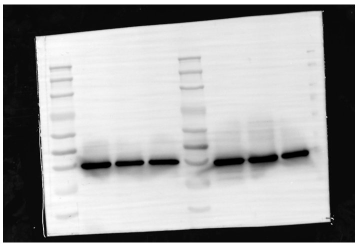

GAPDH

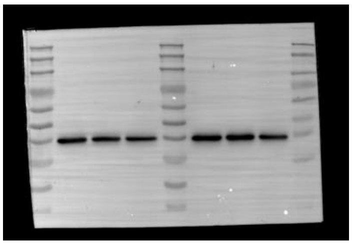

GAPDH

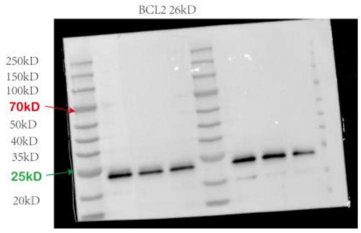

BCL2

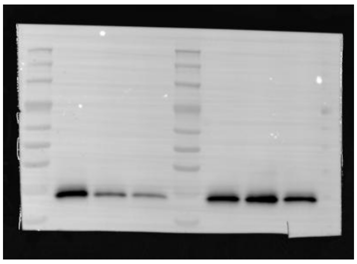

BCL2

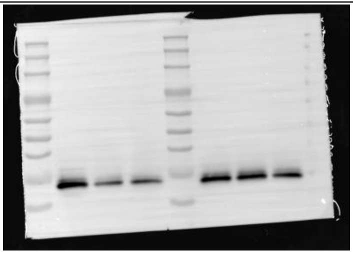

BCL2

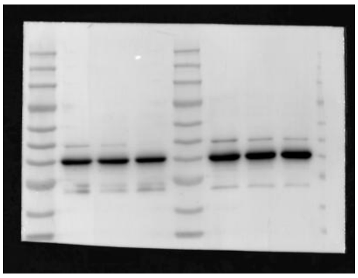

GAPDH

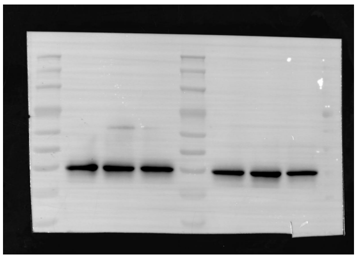

GAPDH

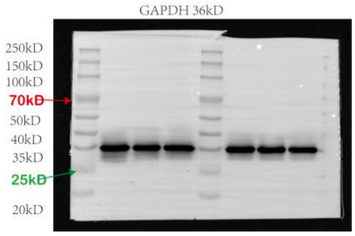

GAPDH

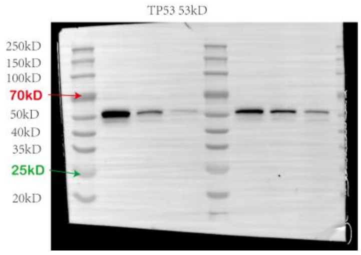

TP53

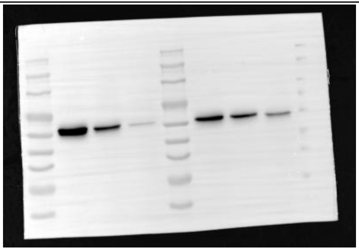

TP53

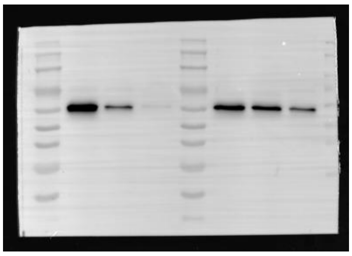

TP53

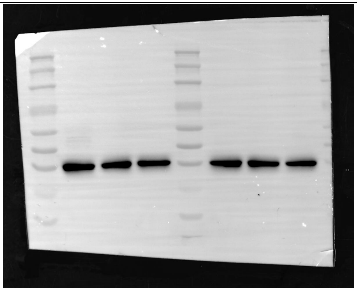

GAPDH

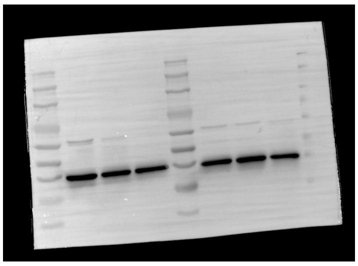

GAPDH

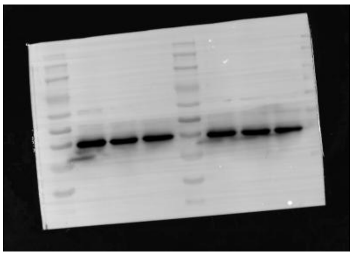

GAPDH

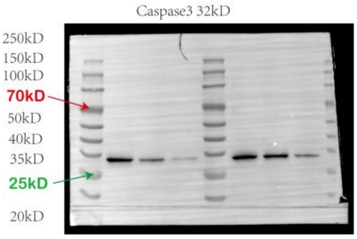

Caspase3

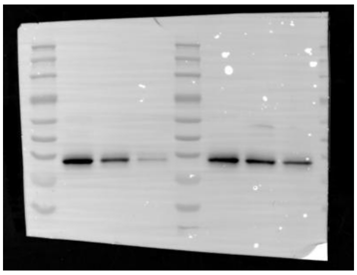

Caspase3

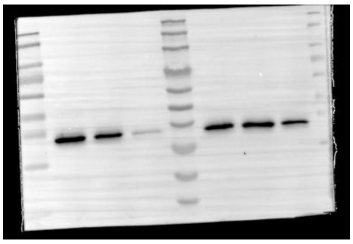

Caspase3

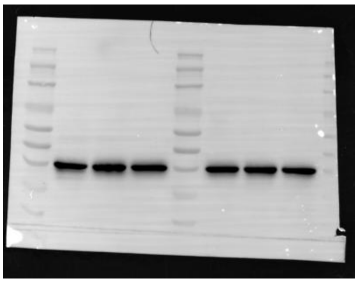

GAPDH

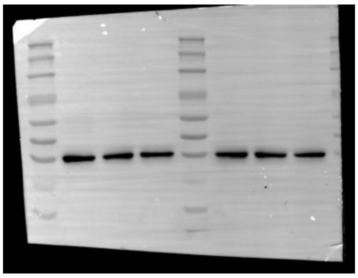

GAPDH

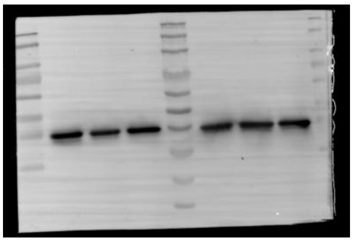

GAPDH

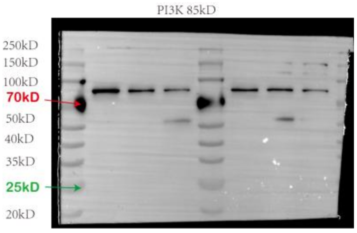

PI3K

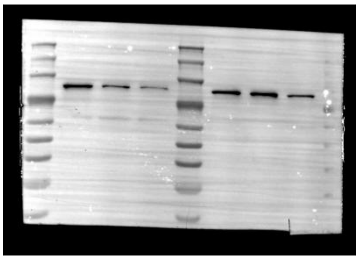

PI3K

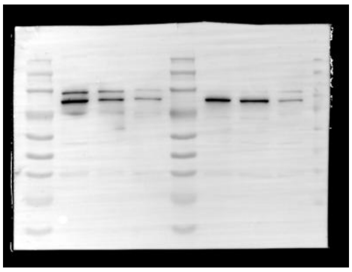

PI3K

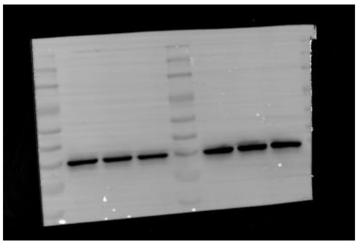

GAPDH

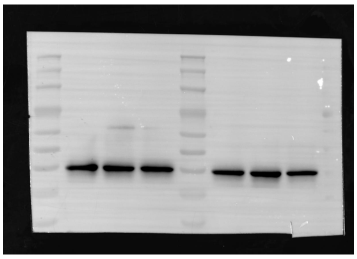

GAPDH

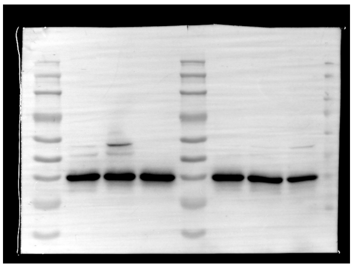

GAPDH

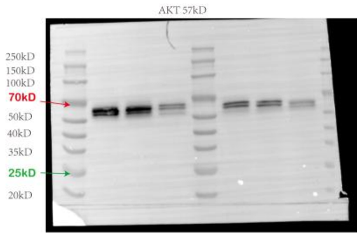	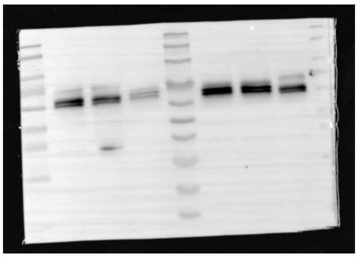	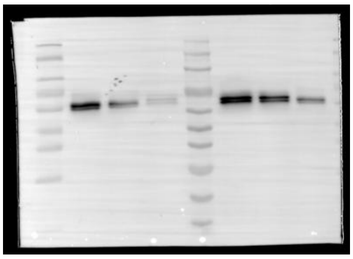
AKT	AKT	AKT
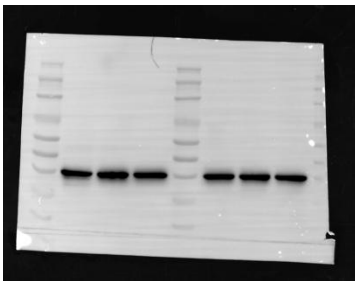	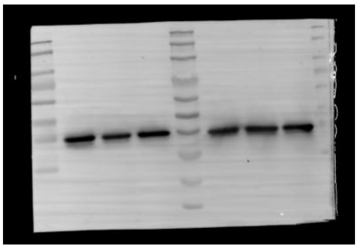	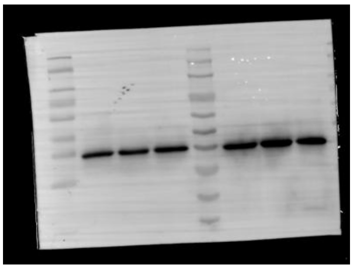
GAPDH	GAPDH	GAPDH
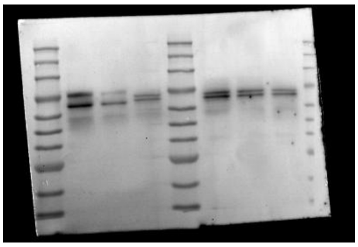	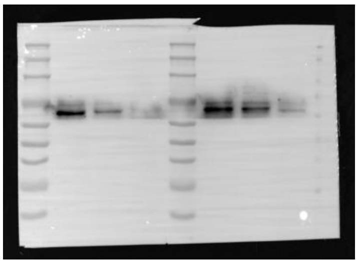	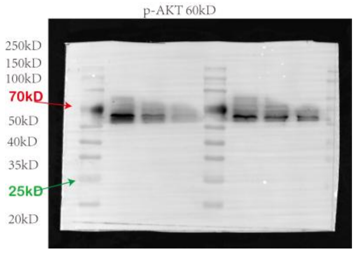
P-AKT	P-AKT	P-AKT
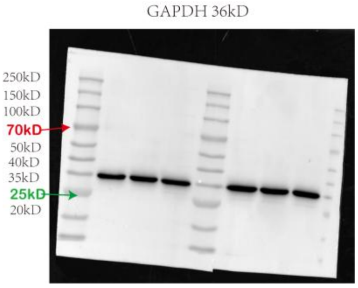	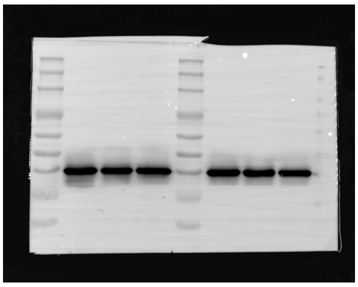	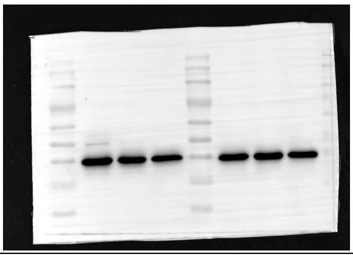
GAPDH	GAPDH	GAPDH
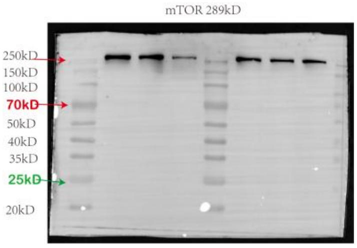	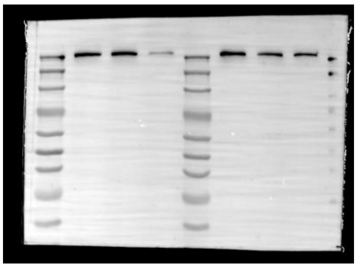	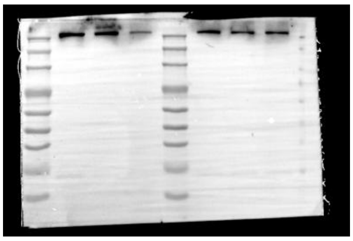
MTOR	MTOR	MTOR

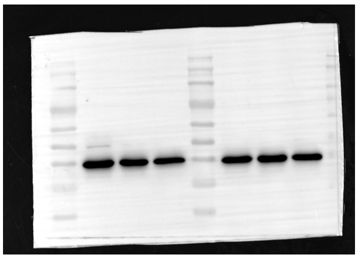	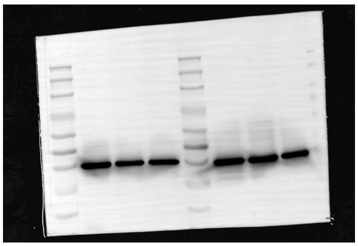	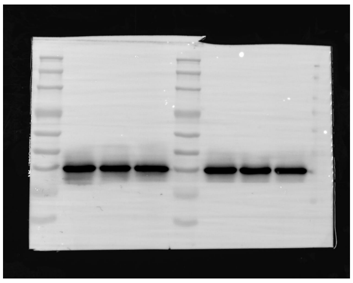
**GAPDH**	**GAPDH**	**GAPDH**
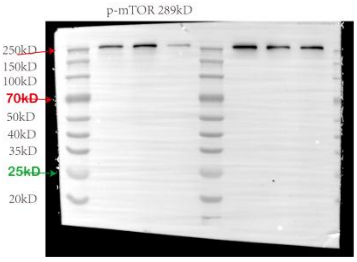	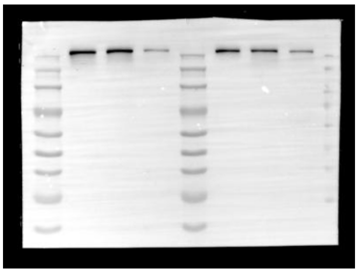	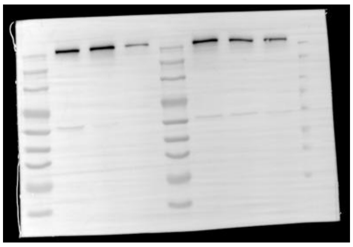
**P-MTOR**	**P-MTOR**	**P-MTOR**
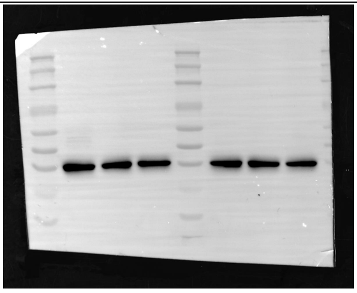	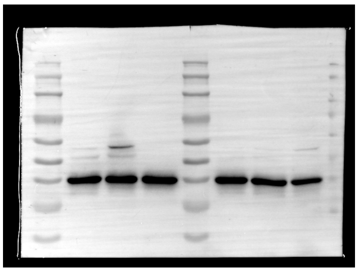	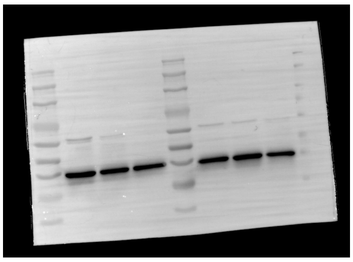
**GAPDH**	**GAPDH**	**GAPDH**
