# Supplementary material for: Targeting HDACs for diffuse large B-cell lymphoma therapy
Source: Sci Rep. 2024 Jan 2;14:289. doi: 10.1038/s41598-023-50956-x (PMC10762105; doi:10.1038/s41598-023-50956-x)
Supplement: Supplementary file 4 — Supplementary Legends. [file 41598_2023_50956_MOESM4_ESM.docx]

**Supplementary Figure 1.** Kaplan–Meier plots show correlations of HDAC expressions with the overall survival of DLBCL patients.

**Supplementary Figure 2.** Heatmaps of the top 50 genes that were positively and negatively correlated with HDACs expression in DLBCL.
